# Juxtaposing Sub-Sahara Africa’s energy poverty and renewable energy potential

**DOI:** 10.1038/s41598-023-38642-4

**Published:** 2023-07-19

**Authors:** Mustapha Mukhtar, Humphrey Adun, Dongsheng Cai, Sandra Obiora, Michael Taiwo, Ting Ni, Dilber Uzun Ozsahin, Olusola Bamisile

**Affiliations:** 1 School of Economics and Management, Guangdong University of Petrochemical Technology, Maoming, 525000 People’s Republic of China; 2 Energy Systems Engineering Department, Cyprus International University, TRNC Mersin 10, Mersin, KKTC Turkey; 3 Operational Research Centre in Healthcare, Near East University, TRNC Mersin 10, 99138 Nicosia, Turkey; 4 College of Nuclear Technology and Automation Engineering, Chengdu University of Technology, 610059 Chengdu, Sichuan People’s Republic of China; 5 School of Management and Economics, University of Electronic Science and Technology of China, Chengdu, Sichuan People’s Republic of China; 6 School of Science, Chrisland University, Abeokuta, Ogun State Nigeria; 7 College of Environmental and Civil Engineering, Chengdu University of Technology, 610059 Chengdu, Sichuan People’s Republic of China; 8 Department of Medical Diagnostic Imaging, College of Health Science, University of Sharjah, 27272 Sharjah, United Arab Emirates

**Keywords:** Environmental sciences, Energy science and technology, Engineering

## Abstract

Recently, the International Energy Agency (IEA) released a comprehensive roadmap for the global energy sector to achieve net-zero emission by 2050. Considering the sizeable share of (Sub-Sahara) Africa in the global population, the attainment of global energy sector net-zero emission is practically impossible without a commitment from African countries. Therefore, it is important to study and analyze feasible/sustainable ways to solve the energy/electricity poverty in Africa. In this paper, the energy poverty in Africa and the high renewable energy (RE) potential are reviewed. Beyond this, the generation of electricity from the abundant RE potential in this region is analyzed in hourly timestep. This study is novel as it proposes a Sub-Sahara Africa (SSA) central grid as one of the fastest/feasible solutions to the energy poverty problem in this region. The integration of a sizeable share of electric vehicles with the proposed central grid is also analyzed. This study aims to determine the RE electricity generation capacities, economic costs, and supply strategies required to balance the projected future electricity demand in SSA. The analysis presented in this study is done considering 2030 and 2040 as the targeted years of implementation. EnergyPLAN simulation program is used to simulate/analyze the generation of electricity for the central grid. The review of the energy poverty in SSA showed that the electricity access of all the countries in this region is less than 100%. The analysis of the proposed central RE grid system is a viable and sustainable option, however, it requires strategic financial planning for its implementation. The cheapest investment cost from all the case scenarios in this study is $298 billion. Considering the use of a single RE technology, wind power systems implementation by 2030 and 2040 are the most feasible options as they have the least economic costs. Overall, the integration of the existing/fossil-fueled power systems with RE technologies for the proposed central grid will be the cheapest/easiest pathway as it requires the least economic costs. While this does not require the integration of storage systems, it will help the SSA countries reduce their electricity sector carbon emission by 56.6% and 61.8% by 2030 and 2040 respectively.

## Introduction

Energy is key to the economic and social development of every country and its global consequence makes it an undeniable subject to be studied from science, economic, engineering, and geopolitical perspectives^1^. Energy has become an indispensable commodity in this globalized age. While the energy crisis in the 1970s led to the development/exploration of alternative sources of energy as well as its corresponding technology, the threat posed by climate changes from greenhouse gases emissions has redirected the focus of energy issues in recent years^2^. Nowadays, the road map to net-zero emission and the integration of more renewable energy (RE) has been highlighted as the priority of many countries^3^. The global sustainable and RE scenario has burgeoned in recent years (although the unprecedented Covid-19 pandemic reduced this growth significantly as different energy policies’ implementation were interrupted)^4^. Renewable energy sources (RES) are now being adopted more in the energy sector of different countries because their benefits are enormous. Nazir et al.^5^ studied the potential impact of wind energy development from a global perspective. They concluded that researchers, stakeholders, developers, and decision-makers should work closely to harness the potential and environmental benefits of wind energy^5^. The International Energy Agency (IEA) has also stated that over 60% of global supply must be from RES by 2050 to have a sustainable environment^6^. Other solutions such as carbon mitigation, carbon emission policies, global energy interconnection, etc. have also been proposed^7^.

While the developed countries and the global world are transitioning into a cleaner and more sustainable energy sector, many developing (especially African) countries are still in abject energy poverty. Research has shown that there is a relationship between electricity access and energy poverty^8^. Based on recent statistics from IEA, about 620 million Africans (two-third of the population) do not have access to electricity while about 730 million use traditional biomass for cooking^6,9^. This existence of energy poverty in a sizable region of the world is a formidable challenge to the proposed global long-term, mid-term, and short-term global carbon emission reduction targets^10^. The slow economic development and extreme poverty experienced in Africa are also related to the continent’s electricity access^11^.

Different scholars have studied the energy poverty that has plagued the African continent, especially the Sub-Sahara region. Batinge et al.^12^ assessed the roadmaps for 100% energy access in the unmet electricity markets in Africa. The study highlighted that the electricity infrastructure in Africa is inadequate for universal electricity access by 2030. Therefore, based on their analysis, private sector funding was suggested as the most viable way to expedite the attainment of 100% access to electricity^12^. Another study^13^ worked on the computation of African’s electricity deficit. A minimum energy poverty line was constructed using field research in urban Nigeria as a case study and 3068 kWh/cap-yr was proposed as the minimum energy required for basic needs in Urban areas. Garba and Bellingham^14^ studied Africa’s energy poverty using Sub-Sahara Africa as the case study. They estimated the impact of solid cooking fuels on the gross domestic product per capita in this region and found that enhanced economic development will influence the household use of solid (traditional) fuels^14^. Szabo et al.^15^ proposed a sustainable energy plan for Africa to leapfrog its energy poverty gap. The methods presented in their study organized the scarcely available energy regional and local geoinformation into maps^15^.

The Sub-Sahara Africa region consists of 46 countries out of the 54 countries in Africa. This region is one of the most deprived of electricity access globally. Its electricity growth capacity in the last 40 years has been half the growth rate of other developing countries/regions^16^. Gregory and Sovacool^17^ proposed the rethink of the governance of energy poverty in Sub-Sahara Africa. In their study^17^, they reviewed three academic perspectives on electricity infrastructure investment. They concluded that 15 distinct issues (including uncommercial tariff regulation, lack of complementary assets, uncertain revenue security, etc.) need to be accommodated if the developmental policy perspective for delivering electricity access to the region will be successful. Another study^18^ evaluated the progress of Sub-Sahara’s electrification as this is a paramount tool to evaluate the energy poverty in the region. The energy sector project finance appraisal documents were used as case studies. They found that, despite the role of financial institution’s development in energy access advancement, the standards that guide these institutions’ actions limit the electrification planning processes^18^. The dynamic relationship/effect of energy-food-water poverty on agricultural sustainability in Sub-Sahara Africa countries was studied by Ilhan Ozturk^19^. It was concluded that agricultural sustainability is a prerequisite for the reduction of the energy-food-water poverty in the region^19^. Similarly, the evaluation of the causes of environmental vulnerability and economic poverty in this region shows that the availability of electricity will drastically reduce the overall poverty rate^20^.

Considering studies on energy poverty for some Sub-Sahara African countries, a recent study^21^ presented a postmodern perspective of energy poverty in Zimbabwe. To construct, understand, and interpret energy poverty in this country, the effect of vandalism of electricity infrastructure, climate change, low utilization of renewables, and sanctions were analyzed. It was concluded that a discursive approach to energy poverty is required to form a balance energy practice and policy^21^. Also, the demand side potentials for reducing energy poverty in South Africa study showed a decline in electricity per capita values^22^. Furthermore, empirical evidence of energy poverty and financial inclusion for Ghana was presented in another research, and energy poverty was reported to reduce by 1% (from 81 to 80%) between 2012 and 2017^23^. Unlike the negative energy poverty rate in Africa, the RE potential in this region is high in comparison to other continents. Based on International Renewable Energy Agency^24^ report, renewables like; solar, wind, biomass, and hydropower have a high unexploited potential in Sub-Sahara Africa.

On the 17th of May 2021, the International Energy Agency (IEA) released a comprehensive roadmap for the global energy sector to achieve net-zero emission by 2050^25^. The transition towards RE and electric vehicles utilization was highlighted as the two keyways in achieving the net-zero emission target. While countries like United Kingdom, China, etc. have also rolled out their plans to achieve net-zero emission in the near future, it is imperative to note that most African countries still lack access to electricity. Although Africa’s population is 16.72% of the world’s population, its electricity generation share in global electricity generation has been around 3% since the early 2000s^26^. Considering the sizeable share of (Sub-Sahara) Africa in the global population, the attainment of global energy sector net-zero emission is practically impossible without a commitment from African countries. Therefore, it is important to start studying and analyzing the feasible/sustainable ways to solve the energy/electricity poverty in Africa. Although studies in literatures have provided some information about the energy poverty situation in Africa, solutions to this energy problems are limited in the existing work of literature. Also, more applicatory studies, analysis, and models are required to explicate the utilization of RE potential in Africa to solve this imminent energy problem in the continent.

Therefore, in this paper, the energy poverty in Africa (specifically Sub-Sahara) is juxtaposed with the high RE potential in this region. Beyond this, the review/analysis of the RE potential and the high energy poverty rate in the continent is presented. Electricity is the most diversified form of energy and therefore it has an integral role in determining the energy poverty of any region. In this study, the generation of electricity from the abundant RE potential in this region is considered from the generation side and presented. In comparison to existing works of literature in the research area, this study is novel as it proposes a Sub-Sahara Africa (SSA) central grid as the way forward and as one of the fastest solutions to the energy poverty problem in this region. Using the EU synchronous grid (that supplies 400 million customers from 24 countries) as a template, the proposed central grid in this study will cut across 12 SSA countries and supply its corresponding customers. Beyond the production of electricity only, the integration of a sizeable share of electric vehicles with the proposed central grid is also analyzed. A model for electricity demand estimation in African countries is also developed in this study. This study aims to determine the RE electricity generation capacities, economic costs, and supply strategies required to balance the projected future electricity demand in SSA. The analysis presented in this study is done considering 2030 and 2040 as the targeted years of implementation. These two years have been chosen considering different global energy targets by 2030 and the IEA 2050 net-zero emission plans.

Based on this proposal, EnergyPLAN simulation program is used to simulate/analyze the generation of electricity from renewables for the central grid while Matlab is used to integrate electric vehicles and hydrogen production (based on the possible excess electricity from the grid). This study will serve as the first step in the right direction for SSA countries to simultaneously transition towards a net-zero emission future and solve their energy poverty. The method and model presented in this study can also be adopted by individual countries as well as regions or continents as a solution to energy poverty and a pathway to net-zero emission attainment. Unlike previous studies, this research is also novel as the hourly energy demand profile is balanced with the production from different RE power plants based on the RE potential in different SSA countries. Considering the high distribution of wind and solar energy resources in Africa, these two resources are the only RE resources considered within the scope of this study. Biomass energy is not considered because of the technicality required for the large installation of biomass power plants. However, pumped hydro storage system is considered as the only storage mechanism in this study. Also, onshore wind power, photovoltaic (PV), and concentrated solar power (CSP) are the three RE technologies adopted in this study due to their popularity/viability in this region (according to existing works of literature). Also, the singular use, as well as the hybrid of the aforementioned technologies, are considered with storage systems. Finally, the hybrid of these technologies and the integration of the import of electricity from the existing power system is also analyzed. The rest of this paper is arranged as follow; the energy poverty and RE potential of the Sub-Sahara African countries considered in this study are explicated in Section "Materials and methods", the materials and methods used in the analysis of the proposed central grid are presented in Section "Results and discussions" while the results from this analysis are discussed in the subsequent section (Section "Conclusions"). This paper is summarized in Sect. 5 by highlighting the outstanding conclusions.

## Sub-Sahara Africa’s energy poverty and renewable energy potential

Despite the rich RE resources in Africa, the continent is characterized by the non-availability of electricity and the lack of access to basic electricity infrastructure^27^. Most of the countries in Africa are heavily reliant on non-RE resources for electricity production. Oil and gas are the predominant energy resources used for energy production in North Africa. The Sub-Saharan region is also reliant on fossil fuels, accounting for about 70–90% of the primary energy supply^28^. Hence, coal, oil, and natural gas are used to meet the fast-growing demand for energy on the continent^29^. Despite the dominating use of these conventional sources for electricity generation, most of these countries are still considered energy-poor nations. According to literature^30^, 69% of the African population makes use of biomass for cooking.

**Figure 1:**
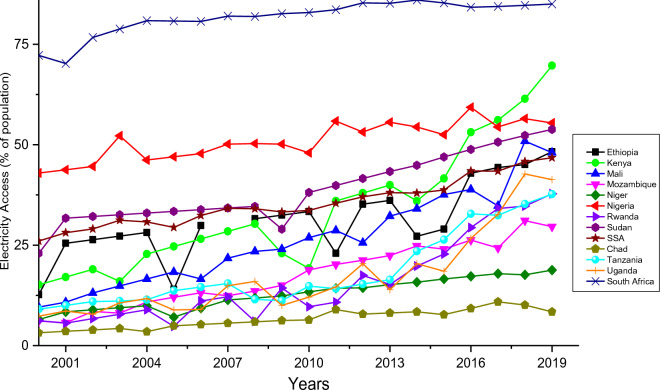
Access to electricity for SSA countries considered^32^.

In this section, the energy poverty level and the RE potential in SSA countries are reviewed and explicated. The energy poverty/RE potential of some selected countries is presented in this section to highlight the imminent energy problem/potency of this region. Based on reports, about 48% of sub-Saharan Africa (SSA) (600 million people) are without access to electricity. The percentage electricity access trend for twelve different countries in SSA as well as a sum for the SSA region in 20 years is illustrated in Fig. 1. It shows that there is 47.6% access to electricity in the SSA region. Several barriers to electrification in SSA ranges from cost barrier to grid connection, socio-economic reasons, corruption amongst electricity providers, high cost of connection, and informal housing challenges^31^. Although the growth over the years in these countries is enormous (Table 1), it is noteworthy that there is still a below-average (50%) access to electricity in the region, with exception of Kenya and South Africa.

**Table 1:** Percentage increase in access to electricity^32^.

Countries	2000	2018	% Increase
Ethiopia	12.700	44.990	254.173
Kenya	15.364	75.000	338.132
Mali	9.379	50.900	442.683
Mozambique	5.982	31.100	419.929
Nigeria	42.856	56.500	31.837
Rwanda	6.200	34.717	459.945
Togo	16.956	51.345	202.811
Tanzania	9.588	35.559	270.879
Uganda	7.738	42.650	451.145
South Africa	71.751	91.230	27.148
SSA (excluding high income)	25.846	47.603	84.183
SSA (IDA, IBRD)	25.995	47.666	83.366

Different solutions have been proposed to the evident energy poverty and problems in the energy sector in Africa. Some selected outstanding literature that have considered different solutions to the energy problems in SSA countries are summarized in Table 2. The research definition/problem statement, methodology, and summary of these studies are highlighted in the tabe. Based on the reviewed studies and other existing works of literature, it is evident that the proposed approach (to solve the energy poverty and utilize RE potential in SSA) in this study is yet to be considered. Hence, the need for the proposed RE-based central grid development in this study.

**Table 2:** Review of literature on energy poverty and RE potential in SUB-Saharan countries.

Country	Introduction/problem statement	Research definition/methodology	Summary
Mozambique	Electricity access focused on the urban regions in Mozambique, with only 5.7% electricity access in the rural areas. This study investigated the possibility of utilizing RE for rural mini/off-grid power supply^33^	The study assessed the use of RE for electricity considering the environmental, socio-cultural, economic, and institutional dimensions. The methods used were data collection, literature surveys, and interviews	Their study concluded that the most viable RE option for rural electrification was small-scale hydropower. The viability of this would be strengthened by institutional coordination
	There exists a huge gap between electricity supply and demand in Mozambique. Their study investigated the energy demand of rural areas, and how small hydropower energy can be used in meeting the energy needs^34^	Semi-structured interviews were supplemented with initial literature research and qualitative document analysis. The methodology was used in examining the links between electrification and sustainability goals based on in-depth literature studies on sustainability. The proposed method in this study was used to establish a nexus approach for water-energy food to promote sustainable development	The study concluded that rural households can afford about 8 – 189 $/MWh of generated hydropower. Also, it was highlighted that the economic feasibility of small hydropower production will improve if small industries are constituted in the region
Ethiopia	In Ethiopia, only 46% of the 110 million inhabitants use electricity for day-to-day activities. Citizens (80%) in the country's rural areas have no reliable energy supply. The energy poverty in Ethiopia was investigated in this study^35^	Direct interviews were used to gather data on energy shortages from the community in both rural and urban locations. The impact of data from the country's central statistical office on households, education, health, and agricultural development was examined. The energy poverty level/effect was clearly defined and compared in Ethiopia's rural and urban areas utilizing statistical data analysis. The quantitative data analysis includes survey data	Energy poverty affects the quality and delivery of education in rural Ethiopia. In terms of education systems and exchanging knowledge about the pandemic, the pandemic COVID-19 is posing more threat to Ethiopia's energy poverty. Also, their study concluded that energy scarcity has a greater impact on Ethiopia's rural areas than it does on its urban areas. Utilization of RES by the government and private sectors was proposed as a viable solution to the energy scarcity experienced by the citizens
	Ethiopia is reliant on importing solar and wind energy technology to meet local demands due to a lack of technical skillset in locally producing RE technologies. Their study analyzed the challenges in exploiting RE sources for electricity production^36^	Parts of the data and information utilized in this paper were gathered from official publications. Semi-structured interviews were supplemented with initial literature surveys and qualitative document analysis. Questions were answered in each interview to highlight institutional and organizational conditions, economic factors, environmental factors, and socio-cultural characteristics. The methodology used to examine the links between electrification and sustainability goals was informed by more in-depth literature studies on sustainability. In total, 50 face-to-face interviews with representatives from mixed-electrified/non-electrified households were done in order to better understand the local sustainability of various power alternatives in the area	It was stated conclusively that because of lack of investment resources, poorly established solar and wind supply chain, trained workforce, and other factors, the region's ability to develop and manufacture solar and wind energy technologies is severely limited. The study suggested that policies that would increase financial incentives for research and development should be prioritized in Ethiopia
South Africa	Despite the vast energy resources, South Africa is facing challenges in sustainable energy development. The prevalence of this problem has caused an increase in greenhouse gas emissions, deforestation, and environmental degradation^37^	Their study investigated the potential of four RES (hydropower, solar, wind, and biomass), and the energy policies in South Africa (alongside Nigeria, and Egypt). The method used was the survey of existing work of literatures on the energy situation in South Africa	Their study concluded that South Africa had the best model for resolving the energy challenges in the region (as compared to Nigeria, and Egypt). It also emphasized the need for energy storage technologies in the country and the need for more efficient energy policies
	As the world's seventh-largest coal producer, South Africa generates roughly 77% of its electricity from coal, resulting in significant environmental degradation. South Africa has the highest per capita greenhouse gas emissions in Africa. Furthermore, due to a lack of distribution networks, centrally generated electricity is unable to reach remote areas^38^	The implementation of solar and wind energy in South Africa was investigated. Data collected from literature survey was used for the analysis	Their study conclusively stated several issues that must be tackled to improve RE production in South Africa. These include political volatility, differing energy strategies, grid infrastructures, and technical skills. Their study also stated that the favorable factors in South Africa which can lead to high RE integration in the energy mixes are reduced price of RE and subsidy
Rwanda	The study analyzed the current RES and RE technologies in Rwanda. It also estimated their potential, as well as addressing the challenges that new and existing RE technologies are facing^39^	To gather data and information for the report, their study used literature survey, analyses, site visits, etc. including a visit to the Rwanda National Electricity Control Centre. Some data were gathered from REG installations across the country	From their data analysis, it wasmstated that 11.33% of Rwandan households are off-grid, while 35.13% are connected to the grid. The government intends to expand off-grid connectivity to reach 100% connectivity by 2024. The study also concluded that households in rural areas that are far from the national grid or who use inadequate electricity will be encouraged to use off-grid solutions such as mobisol, mini-grids, or solar PV solutions to gain access
	To ensure that the (48% Rwandans without access to electricity in the) country has access to cheap and sustainable electricity, Rwanda will need to strengthen its energy sector with projects, policies, and private partners^40^	To gather data and information for their study, their research used a literature research, analyses, site visits, and a survey to the Rwanda National Electricity Control Centre, as well as some data acquired from REG installations across the country. The study proposed a policy and semi-private operator model in which solar-powered mini-grids and smart metering systems can provide a long-term solution to the energy crisis by rising electricity reliability and supplying power to various electricity consumers	Their study concluded that solar-powered mini-grids can help with energy access for rural areas, industries, energy efficiency, and climate change mitigation, which is an important tool for reducing carbon emissions
Mali	Carbon emissions from increased energy consumption have become a major policy issue in Sub-Saharan Africa, especially in Mali^41^	The relationship between biomass energy use, economic development, and carbon emissions in West Africa from 1980 to 2010 was examined in their study. Furthermore, a three-stage least squares model to estimate a simultaneous equation model (3SLS) was used	Overall, their findings show that GDP, biomass use, and carbon emissions in Mali (alongside Nigeria, Burkina Faso, Gambia, and Togo) have a full and important interactive relationship
Kenya	Kenya lacks a national rural electrification program, which is a critical component for policymakers to set policy direction and establish a program roadmap for energy access^42^	Their study conducts a thorough spatial mapping of Kenya's current energy infrastructure and develops a rural electrification spatial model for Kenya (RE RU KE tool) to define optimal strategies for the various locations, taking into account the current energy status and local resources. To electrify remote areas in Kenya at the lowest possible cost, the model considers the potential of traditional approaches (diesel gensets), renewable technologies, hybrid systems, and the possibility of central grid extension. Their paper focuses on a developed geospatial/numerical methodological approach for rural populations who are still without electricity	RE RU KE's findings highlight the importance of updating the national strategy and focusing on a comprehensive scaling-up of RE to electrify off-grid areas. At the same time, the model emphasizes the potential for solar home systems to increase universal access to basic electricity
Tanzania	Tanzania is looking to increase its RE production by emulating various methods of ensuring sustainable and usable energy supply to its socio-economic and political sectors^43^	This study gives a literature survey analysis of the state of sustainable energy development in Tanzania. The methodology used is data from related studies	It was conclusively stated that Tanzania has a high RE growth potential. However, due to various socio-economic and political factors, research efforts have primarily focused on hydropower projects, and other RES are underutilized. Their study suggested that a viable market condition should be created for RE investors
Nigeria	There are more than 80 million Nigerians who do not have access to electricity. The high percentage of the population of these countries live in rural areas and below the poverty line contributes to the lack of access to electricity^44^	The chronic failure of public infrastructure such as healthcare, education, and protection to contribute to the country's weak electricity generation, transmission, and distribution capacity is analyzed using the desktop method and empirical formulas, as well as the current government's commitment to bolstering our generation capacity and electricity access through policies and investment	Their findings showed that there is an urgent need for a smart deployment of distributed generation units to promote and facilitate the economy's ongoing expansion, as well as the need for a long-term road map that integrates the success of countries facing similar challenges
	For decades, electricity production, transmission, and distribution have been a major challenge in Nigeria. Approximately 41% of the country's population currently lacks access to electricity^45^	Their study proposes an economically feasible, green, and long-term strategy for Nigeria to achieve 100% electrification by 2030. Energy PLAN was used to perform a one-year study focused on hourly time-steps, which used different combinations of energy technologies	They forecasted Nigeria's electricity demand to be 200 TWh/yr by 2030. If a single power technology is used, a 36,000 MW natural gas capacity will be needed to meet this demand. To satisfy the electricity demand, the most sustainable plan is to use a combination of natural gas and photovoltaic or natural gas and onshore wind

### Review of SSA power portfolio

The future of power portfolios and the integration potential of renewable energy (RE) in Sub-Saharan African (SSA) nations have been the subject of extensive research, reflecting the increasing significance of sustainable energy development in the region. Numerous case studies have examined various facets of this topic, casting light on the opportunities and challenges of the energy transition in SSA. These studies emphasise the vast solar, wind, hydro, and geothermal renewable energy resources available in SSA. They highlight the significant potential for integrating RE into the power portfolios of SSA nations, thereby facilitating a transition towards cleaner and more sustainable energy systems. Furthermore, some studies have assessed the challenges regarding the renewable energy transition and more specifically, grid instability in SSA. The study by Jose et al.^46^ emphasized the role of hydropower in expanding intermittent renewable in Ghana by 38%, due to the flexibility of the resource. Their study considered three strategies for using hydropower to augment the intermittent renewable energy in Ghana: solar, wind and solar with storage, and reoperation of the Akosombi hydropower plant. Their study went further to discuss the harmful consequence of hydropower on aquatic ecosystems and cross-sectoral water conflicts.

Higher penetration of variable renewable energy (VRE) to meet the 10% RE target by 2030 highlighted in the Renewable energy master plan of Ghana was analyzed in the study by Ampah et al.^47^. They proposed a high renewal penetration (HREP) which includes power-to-X, vehicle-to-grid, and pumped hydro storage to maximize the VRES. Their result showed a penetration of about 26%, and 30% of solar and wind in the total electricity generation of 2030, as against 2.3% and 1.7% in the business-as-usual scenario. The study by Saadi et al.^48^ considered regional trade to promote renewable energy in the SSA region. This regional initiative is being promoted by the African Clean Energy Corridor (ACEC). In their study, they found that when regional electricity trade was implemented as against a business-as-usual (BAU) of increasing fossil fuel power, it resulted in lower system cost and higher penetration of wind in Kenya and Ethiopia. This facilitated emission reduction by 40%, relative to the BAU scenario of only hydropower to meet residential demands. Technical analysis for both local and national electricity planning for Senegal was carried out in a study by Sanoh et al.^49^. Their study specifically considered the cost drivers for electrification, to simultaneously improve access to basic infrastructure and drive down cost. Their analysis showed that the electrification of rural areas will depend on demand: when grid costs are reduced by half relative to the base scenario, grid connection becomes more favourable for rural electrification. More similar reviews of power system approaches can be retrieved from^50^.

The energy system in SSA requires urgent attention if the region is serious about improving electricity access and solving poverty. The energy security of the region also begs for diversification from the conventional fossil fuel-based energy systems. Transitioning to renewable energy production is more of a policy bottleneck, rather than the availability of resources, as the region is blessed with abundant solar, wind, biomass, and hydropower resources. Some studies have suggested several technical solutions like grid extension, decentralized systems, and other country-specific efforts. Though these solutions will play a huge role in transforming the energy situation in SSA, and in cutting emissions, more approaches can be taken. In this work, the authors have suggested a centralized grid system to promote interconnections among the SSA region. A centralized and regulated system of import and export of energy systems among countries is an approach that is also backed by the DESERTEC organization in the European context^51^. To put an argument for this innovative grid system in the African region, a detailed analysis needs to be done regarding the technical and economic feasibility, as well as the environmental implication. We believe that this study will serve as a useful instalment towards achieving higher RE penetration and energy security in SSA.

## Materials and methods

**Figure 2:**
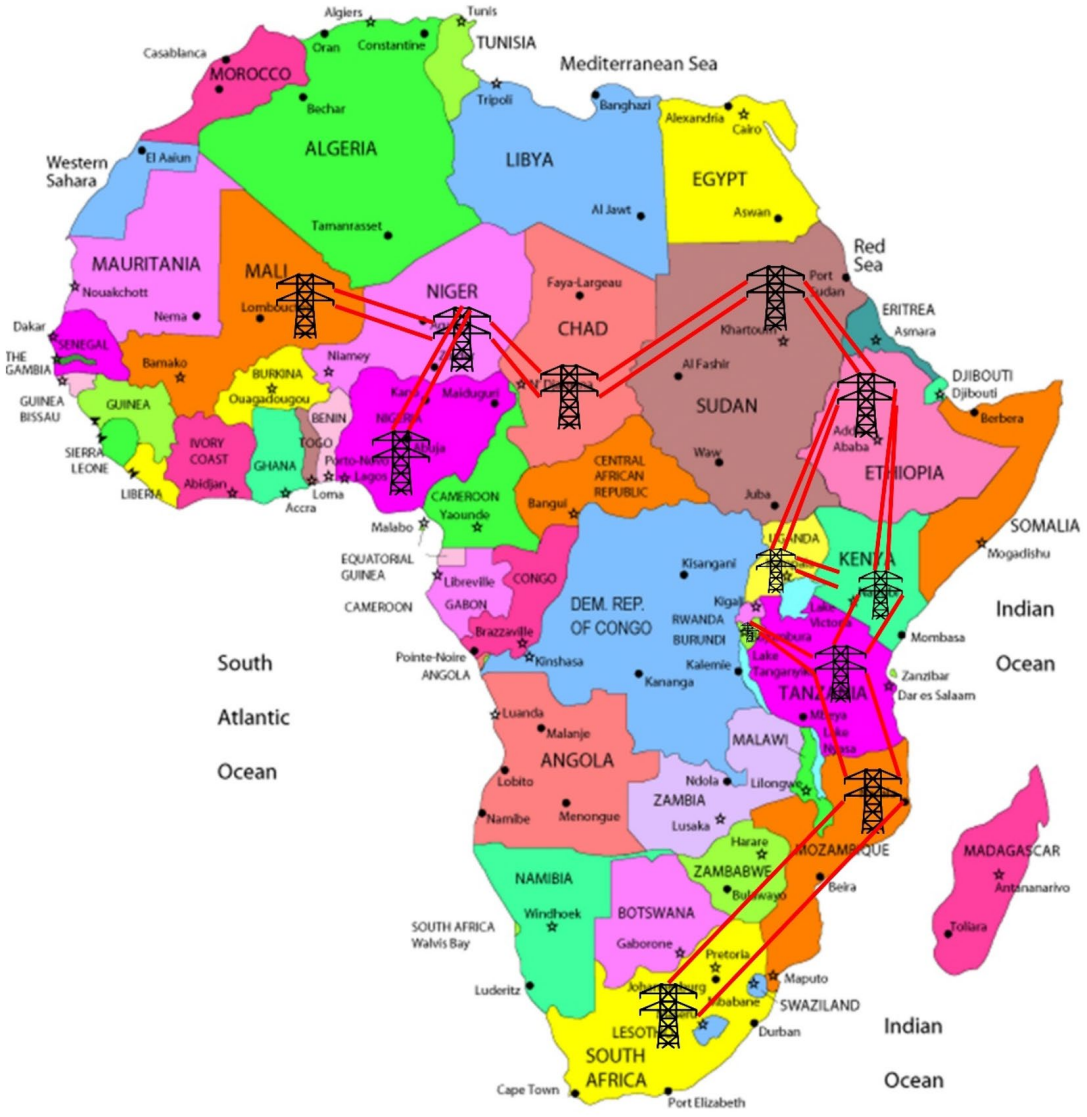
Selected African countries and proposed central grid layout.

In this section, the methods and materials adopted for the analysis are justified. EnergyPLAN simulation that is used for the analysis of the RE-integration is also briefly introduced. It is noteworthy that the analysis presented in this study is conducted on an hourly time step. Twelve different countries (Fig. 2) have been chosen for power supply and the RE potential of these countries as well as their electricity demand has been used in this analysis. The countries have been selected based on data availability, proximity to other countries with sufficient data, land size, and economic status within SSA.

In summary, these countries are selected to follow the grid line (highlighted in Fig. 2) and also to enhance easy expansion in the future. Considering the recent targets set by different countries/organizations for the world at large to achieve a high RE penetration, the years 2030 and 2040 are the targeted years of implementation of the proposed models in this study.

### Research modeling and definitions

**Figure 3:**
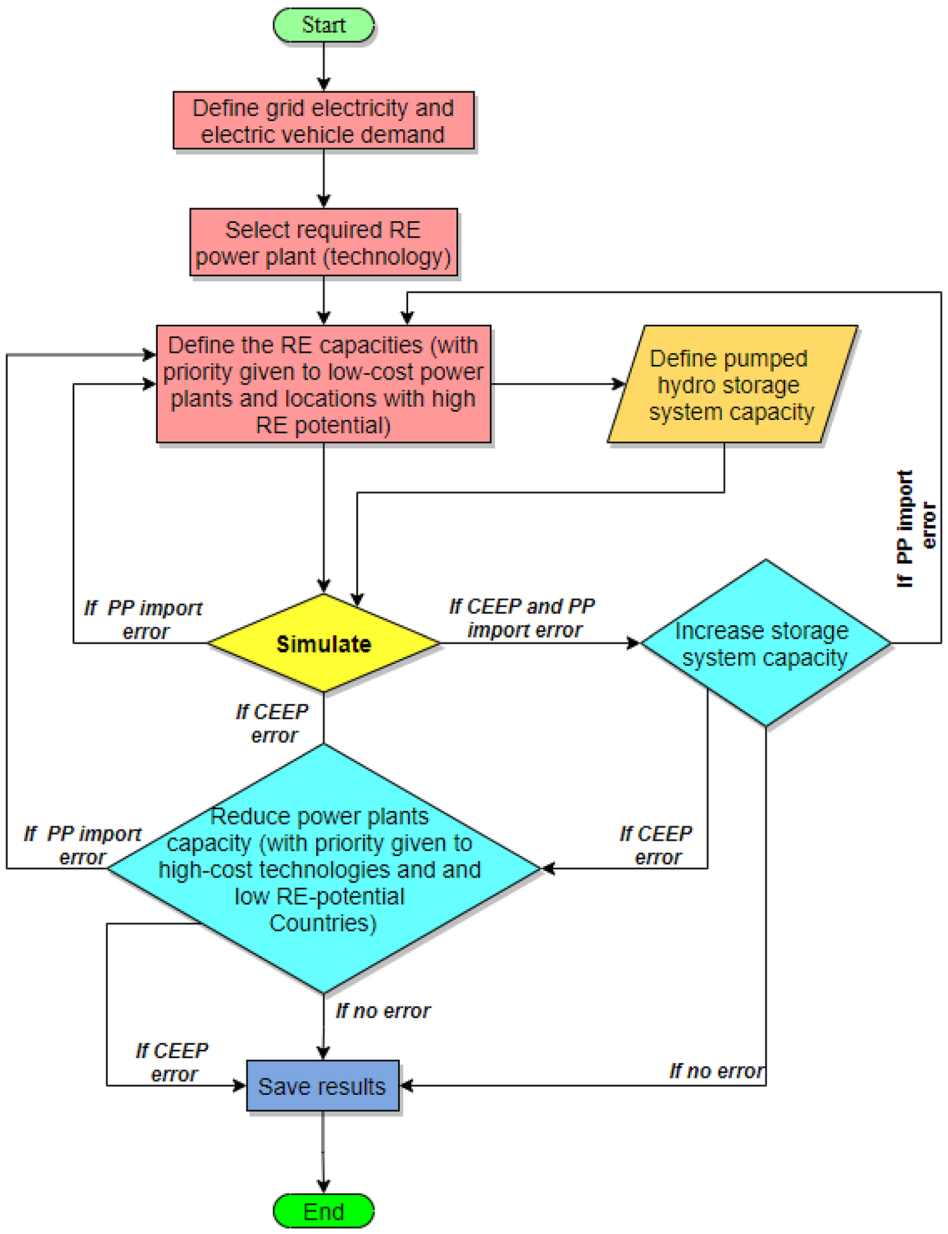
Research model summary.

**Figure 4:**
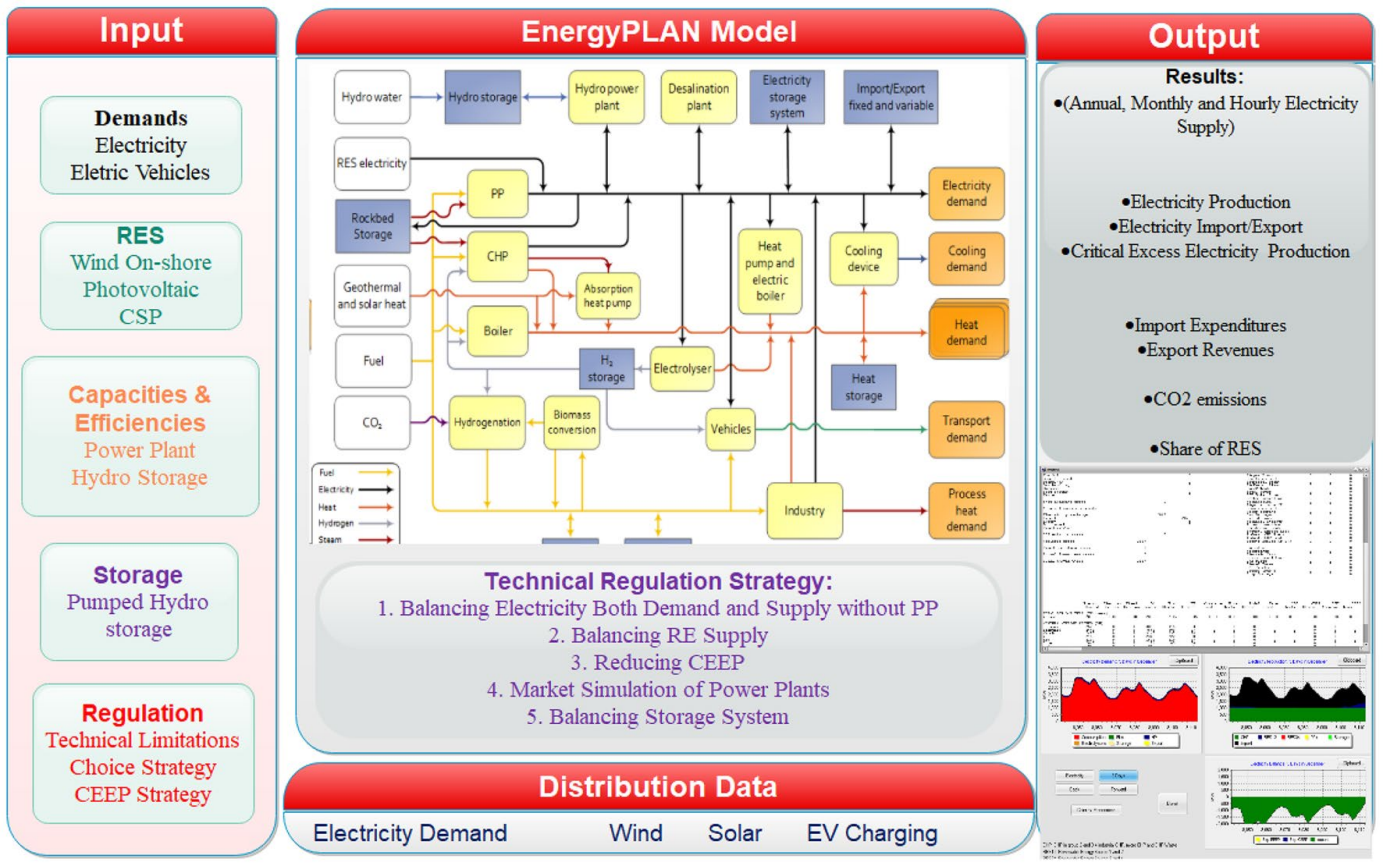
Schematic diagram of research model.

One of the main challenges attached to the use of solar and wind energy for electricity production is the stochastic and intermittent nature of these energy resources. Therefore, it is imperative to create a model that can work beyond this barrier. Solar PV, CSP, and onshore turbines are the RE technologies considered in this study while pumped hydro storage is used as storage due to the hydropower potential in the SSA region. The model used in estimating the required generation capacities to abate the energy poverty in the selected 12 SSA countries is summarized in Fig. 3. Since EnergyPLAN is used as the simulation platform for the proposed model, the schematic diagram of the whole model is presented in Fig. 4.

EnergPLAN simulation tool is a data-intensive simulation program, therefore, the electricity demand profile, RE-technology production profile, EV demand profile, etc. are required. Based on the defined electricity demand, the RE-technology (as well as the production profile for the RE-technologies in each country) is selected and the capacities are simulated to meet the estimated demand. For the simulation program, there are four different outcomes displaced after each simulation. These include.

- ***CEEP warning*** This implies the availability of critical excess electricity production (CEEP) in the proposed grid, and this is caused by the high integration of RES. In this study, the CEEP is reduced to the minimum, however, it cannot be eradicated for most of the scenarios. Therefore, the production of hydrogen from the CEEP is also analyzed.
- ***PPI warning*** The grid electricity demand is higher than the power production. For this case, it is paramount to increase the power plant capacities to eradicate this warning.
- ***No CEEP and PPI warning***: This reflects a balanced electricity demand/supply scenario.
- ***CEEP and PPI warning*** In this scenario, there exists a gap in the electricity demand/supply as some of the demands for some particular hours are not met. However, there is also high-RES integration leading to the production of CEEP. It is paramount to eradicate the PPI warning if this occurs during the simulation process.

The model is built such that there is no power production import (PPI) error and to reduce CEEP. In this study, 12 different models are developed, and six different scenarios are considered for the years 2030 and 2040 respectively. These include.

- ***PV*** This case scenario considers the singular use of solar PV and pumped hydro storage to meet the electricity demand for 2030 and 2040.
- ***CSP*** The singular use of CSP and storage is considered in these case scenarios.
- ***Wind*** Onshore wind power is integrated with storage in this case scenario for targeted years.
- ***Hybrid with low storage*** For this case scenario, the hybrid of all the RE technologies is simulated considering the minimal storage requirement.
- ***Hybrid with high storage*** The pumped hydro storage is prioritized in this simulation and the model is simulated to maximize storage with minimal RE technologies requirements.
- ***Hybrid with Iiport power*** To incorporate the existing power systems and other fossil-fueled plants with the proposed RE central grid in these countries, this scenario considers the import of electricity from other power plants instead of storage (to balance the RE electricity supply).

The input parameters used in this study including electricity demand profile, total electricity demand estimation, EV profile, RE-profile and power production, and hydrogen production are explained in Section "Hybrid RE power technologies". This research is based on the following assumptions and technical strategies:

- Power system/network faults are neglected; therefore, the power plants are assumed to be available throughout the year.
- The distribution network and transmission line capacity are sufficient for the electricity produced.
- The RE powerplant capacities are defined following the capacity factors and the land size of each country and the SSA region.
- Financial constraints or cost limitations are neglect for the power plants’ capacities modeling.
- The EnergyPLAN only allows the integration of seven RE technologies at once, therefore, seven countries are considered for each case scenario presented in this study.
- The analysis is done based on a constant load profile.

Just like other hybrid energy simulation platforms, EnergyPLAN has some limitations and the two most relevant limitations to this study are:

- There is only provision for the integration of wind onshore, solar PV, and CSP technologies for a maximum of seven different locations, therefore, seven countries are considered for electricity production into the grid for each case scenario.
- The pumped hydro storage system is also limited to input spaces; hence, two separate pumped hydro storage systems are used to balance the systems.

### RE integration and input parameters

Since the EnergyPLAN is an input/output program, the data used for the simulation presented in this study is defined in this section. Also, the mathematical models used in estimating the RE technologies’ production and the electricity demand are highlighted.

#### Estimated electricity demand

One of the main challenges with energy-related studies in SSA is the unavailability of data for accurate estimations/predictions. The electricity access ratio (Fig. 1) in all the countries in SSA is less than 100%^32^, therefore, the electricity demand data available are based on the percentage access to electricity in different countries. To estimate the electricity demand in these countries by 2030 (with exception of Nigeria), a mathematical model (Eq. (1)) is created with reference to the electricity consumption in individual countries in 2018.

1 $${TED}_{\mathrm{2018,1}}=\frac{{EC}_{ \mathrm{2018,1}}}{{\%Elec}_{access}}$$

where ${TED}_{\mathrm{2018,1}}$ is the expected total electricity demand (in TWh) in 2018 for a particular country labeled as $1$ considering the entire population of that country. ${\%Elec}_{access}$ is the percentage of the total population with access to electricity in 2018 and ${EC}_{ \mathrm{2018,1}}$ is the reported total electricity consumption in 2018 (in TWh). These two parameters were extracted from the International Energy Agency Report^32^. Then the total electricity demand for the next year (${TED}_{\mathrm{2019,1}}$) can be estimated as:

2 $${TED}_{\mathrm{2019,1}}={TED}_{\mathrm{2018,1}}\cdot b$$

where $b$ is the yearly electricity demand proportional increase. In this study, $b$ is taken as 1.015 for South Africa and 1.05 for the other countries i.e., 1.5% and 5% increase in total electricity demand yearly for South Africa and other SSA countries respectively. It is noteworthy that South Africa has one of the best electrical systems and the highest percentage access out of all the SSA countries, hence the 1.5% increase in demand considered in this study. Therefore, the electricity demand by 2030 can be estimated with Eq. (3).

3 $${TED}_{2030, 1}=\frac{{TED}_{2029, 1}\cdot b \times {TED}_{2028, 1}\cdot b \times \dots \dots \dots \dots \dots ..\dots \dots ..\times {TED}_{2018, 1}\cdot b }{ {TED}_{2028, 1}\cdot b \times { TED}_{2027, 1}\cdot b \times \dots \dots \dots \dots \dots ..\times {TED}_{2018, 1}\cdot b}+\varepsilon$$

Assuming $n={TED}_{\mathrm{2029,1}}\cdot b$, then Eq. (3) can be simplified and presented in a factorial form as:

4 $${TED}_{2030, 1}=\frac{ n! }{ \left(n-1\right)!}+\varepsilon$$

**Figure 5:**
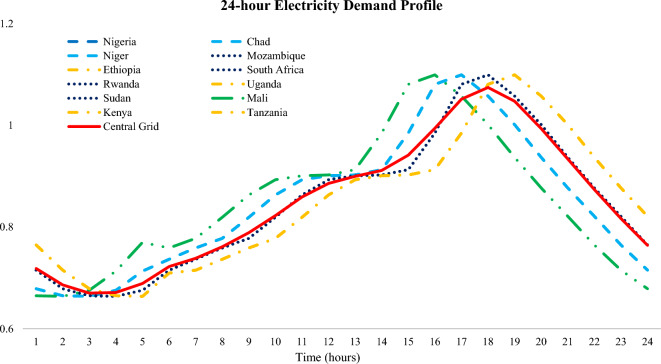
Central grid electricity profile estimation.

**Figure 6:**
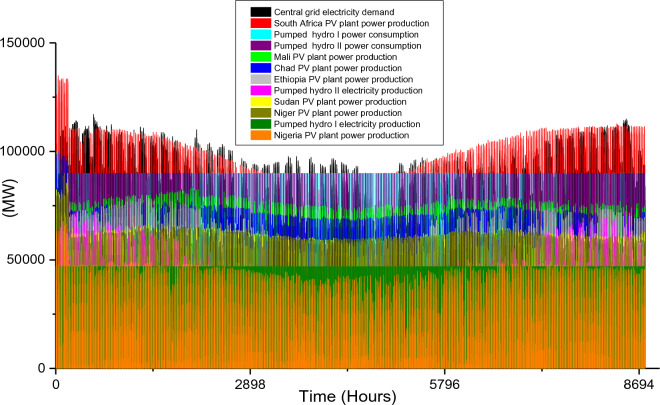
Solar PV case scenario electricity demand and supply for year 2030.

It is noteworthy that $\varepsilon$ is an uncertainty term used in adjusting the estimated total electricity demand by 2030 to suit the individual countries considering their expected population growth and energy sector development (Figs. 5, 6).

Considering the eleven countries (excluding Nigeria) with a label/subscript of 1, 2, 3, ……, 11, the estimated electricity demand for all the countries in this research is calculated with Eq. (5).

5 $${TED}_{2030}=\sum {TED}_{2030, 1}+{TED}_{2030, 2}+ \dots \dots \dots +{TED}_{2030, 11}+200$$

According to research in literature^52^, the electricity demand for Nigeria is estimated to be 200 TWh by 2030 hence the introduction of the term “200” in Eq. (5). The total electricity demand for all the 12 countries by 2040 is estimated considering a 12.1% increase from the 2030 total electricity demand. Since the analysis presented in this study is on an hourly time-step, it is paramount to accurately distribute the estimated electricity demand considering the hourly profile. In the selected 12 countries, there exist four different time zones (as seen in Fig. 12), therefore the typical hourly electricity demand profile in EnergyPLAN is adjusted such the proposed central grid demand profile considers the variation in time zones (Fig. 5).

#### RE technologies modeling

Due to the lack of data in SSA, there may be difficulties in estimating the exact RE technologies production of the proposed power plants. However, there is a provision on the EnergyPLAN simulation program to solve this problem. The simulation program shows the capacity factor of defined systems. Therefore, the capacity factors of all the locations (countries) considered for Solar PV, CSP, and onshore wind power plant installation have been extracted from literature and are used to adjust the production of these powerplants. Ditto, an actual estimation can be made accordingly. A summary of the capacity factors and their corresponding references are presented in Table 3.

**Table 3:** Capacity factor.

Country	PV^64–71^	CSP^45,71–73^	Wind^67,71,74–78^
Kenya	–	–	37%
Ethiopia	19.8%	24%	27.48%
Chad	23%	33.5%	26.61%
Mali	20.5%	32%	21.7%
Nigeria	19.2%	25%	15.7%
Sudan	22%	35%	-
Niger	21.9%	32.5%	38.2%
South Africa	25.7%	30.1%	35.4%
Mozambique	–	–	25%
Uganda	–	–	–
Tanzania	–	–	30%
Rwanda	–	–	-

##### Solar PV/CSP

Although most countries in SSA receive over 2000 kWh of global solar radiation annually, the development of solar energy power plants in this region is extremely small^53^. Considering the impact, applicability, and integration of PV/CSP technologies in global energy production, these two solar-based technologies are the only ones considered in this study. Due to the technical limitation of the simulation program used in this study, seven countries are considered for the modeling of CSP and PV power plants.

These countries include Ethiopia, Chad, Mali, Nigeria, Sudan, Niger, and South Africa. The countries are selected based on data availability, land size, and RE technology potential (based on their capacity factors), as highlighted in Table 3. Due to the huge landmass in SSA, a maximum of 200,000 MW and 100,000 MW of CSP/PV are set for big countries and small countries respectively. The typical meteorological year data^54^ is used to estimate the power production from the CSP and PV power plants for each of the countries. The power production is based on a 1 MW power plant considering all the technical details (including losses) of each technology when in actual operation^55^.

##### Onshore wind

Power production from wind energy has risen to prominence in recent years as onshore and offshore wind power plants dominate this sector. Countries like Denmark^56^, the USA^57^, China^58^, Germany^59^, India^60^, etc. have integrated a huge amount of wind power into their power systems showcasing the integrality of this technology on a larger scale. According to literature^61^, the potential of onshore wind power in SSA and Africa is enormous. According to the International Finance Corporation report, the technical onshore wind resource potential in Africa is over 59,000 GW and this is sufficient to power the continent 250 times^62^. Also, it was stated in another study that the continent only taps 0.01% of its wind power potential at the end of 2020^63^. Ditto, onshore wind power production is explicated considering seven of the twelve as the wind farm location. The wind energy potential for these countries (considering an 80-m wind turbine hub height) has been extracted from the WindPRO software database. The onshore wind power plant production and analysis is based on an actual wind power plant that is embedded in the EnergyPLAN simulation program. This analysis also considers all the technical details and losses in wind power production as well as the intermittency of wind energy.

##### Pumped hydro storage and hydrogen production

Pumped hydro storage is regarded as one of the cleanest energy storage systems and its utilization has emerged in recent years. The good hydropower potential across most of the SSA countries^79^ is also a pointer to the fact that pumped hydro storage is adaptable to this region. This technology constitutes about 160 GW of the power stored globally and its advantages include; less land utilization, high efficiency, low cost, etc.^80^. In this present study, pumped hydro storage is the only storage system considered. Based on the simulation program adopted for this study, two pumped hydro storage systems are defined. Following literature^81^, the efficiency of the pumps and turbines integrated with the storage system are 80% and 90% respectively.

It is inevitable to have CEEP when a grid system is based on RE technologies like solar and wind. Although the use of storage systems has been proffered, studies have shown that it is more cost-effective to switch off the RE-based power plants when the supply is higher than demand. In recent years, the production of hydrogen from electricity has risen to prominence and the use of RE electricity to generate hydrogen through the power-to-gas (PTG) process has a greater/more sustainable advantage^82^. Since this technology is still regarded as a relatively expensive technology, it is proposed as an alternative means of utilizing all the possible CEEP from the central grid system^83^. Specifically, the 0.019 kg/kWh conversion rate published in Nature Energy Journal for the PTG process is adopted in this study^82^.

### Electric vehicle Integration

The transportation sector is one of the core areas for decarbonization policies as it accounts for about 25% of total carbon emissions globally. Electric mobility is one of the proposed solutions for sustainable transportation and greenhouse gases reduction. Many developed and developing countries have adopted the use of electric vehicles to decarbonize their transportation sector. In this study, the integration of a sizable number of electric vehicles is considered and analyzed with the proposed solution. Since the analysis presented in this study considers 2030 and 2040 as the target, it is important to include the prospective development of electric vehicles in SSA. Therefore, a 12-h daily charging profile is implemented in the simulation of the proposed central renewable electricity grid in this study. The electric vehicles are expected to charge typically between 7 p.m. and 7 a.m. daily. This charging profile will further enhance the consumption of wind-based electricity during the night hours, thereby enhancing the stability of the system.

### Greenhouse gas emission reduction potential and economic analysis

Although RE-based power systems offer a sustainable pathway to a cleaner future, the cost of these systems is still a concern in some developing countries, especially in SSA. Therefore, the economic viability of the proposed central grid system is analyzed. So also, the potential greenhouse gases emission reduction. The 2030 cost database that is available on the EnergyPLAN database system is used in this study to determine the total investment cost and the total annual cost (considering the lifespan of the RE-system).

The economic parameters used in analyzing the economic viability of the system are summarized in Table 4. Also, the interest rate used for the economic analysis is 3%. In the economic analysis, the fixed/variable operation and maintenance cost is also considered (Table 4).

**Table 4:** Economic analysis input parameters^84–86^.

Type of power plant	Lifetime (years)	Fixed O&M (% of investment)	Investment cost (M$/unit)	Variable O & M cost ($/MWh-e)
Solar PV (MWe)	40	1	0.82	
CSP (MWe)	25	8.2	5.98	
Onshore Wind (MWe)	25	2.59	1	
Pumped hydro storge (GWh)	50	1.5	7.5	1.19
Turbine for storage system (MWe)	50	1.5	0.6	1.19
Pump for storage system (MWe)	50	1.5	0.6	1.19
***Greenhouse gases emission analysis input parameters***				
Greenhouse gases	#6 fuel oil Emission factors	CO2	0.263 kg/kWh	
		NO_X_	0.743 g/kWh	
		SO_X_	1.47 g/kWh	

Also, the emission factors for #6 fuel oil (with 33% fuel combustion efficiency) are used to determine the greenhouse gas emission reduction potential. The specific emission factors for the #6 fuel oil which is used in some power plants is also summarized in Table 4.

### Mathematical modelling

In the section, the equations used in modeling the RE technologies and making economic analyses are presented. Typically, the design of a solar PV consists of calculating the appropriate number of PV modules that are required to convert solar energy into direct current (DC) power. The output power of each PV module at hour* t (p*_*PV(t)*_*)* can be modeled with Eq. (6):

6 $${p}_{PV}\left(t\right)=I\left(t\right)\times A\times {\eta }_{PV}$$

where I(t) denote the hourly solar insolation (kW/m^2^), η_PV_ is the efficiency of the PV module and* A* denotes the PV module area (m^2^). In this study, losses in PV modules are also considered as this has been pre-program in the EnergyPLAN model.

Unlike solar PV system that involves the direct conversion of solar radiation to electricity, CSP systems convert solar radiation to thermal energy and this thermal energy is used to generate electricity using a power (steam/gas) cycle. This involves different complex thermodynamics processes and it has been presented in literature^87^.

The onshore wind power plant considered in this study is modeled based on a 1 MW actual wind farm. All the technical details (including losses) of a wind power plant have been pre-programmed on the EnergyPLAN simulation platform. In summary, the electricity generated by each wind turbine in the wind farm can be modeled with Eq. (7).

7 $${p}_{WT}\left(t\right)=\left\{\begin{array}{c}0\\ {P}_{r}\\ {P}_{r}\end{array}\right..\frac{{v}^{3}\left(t\right)-{{V}^{3}}_{ci}}{{{V}^{3}}_{r}-{{V}^{3}}_{ci}} \begin{array}{c} v\left(t\right)\le {V}_{ci} or v(t)\ge {V}_{co}\\ {V}_{ci}<v(t)<{V}_{r}\\ {V}_{r}<v(t)<{V}_{co}\end{array}$$

where ${V}_{r}$ is the speed rated velocity, ${P}_{r}$ is the rated power, and ${V}_{co}/{V}_{ci}$ are the cut-out/cut-in wind speed.

The total investment cost (${I}_{tech}$) of each power technology can be modeled by considering the per-unit price (${P}_{unit-tech}$) and the total capacity (${C}_{tech}$) of the technology (Eq. (8)).

8 $${I}_{tech}={C}_{tech}\times {P}_{unit-tech}$$

The total annual cost of each technology/technological combination (${A}_{TOC-tech}$) is modeled (in Eq. (9)) as a function of the investment cost (${A}_{investment-tech}$) in Eq. (10) and the fixed operational cost (${A}_{FOC-tech}$) in Eq. (11). The third parameter in Eq. (9) (${A}_{VC-tech}$) is the variable cost and this is modeled as a percentage of the investment cost.

9 $${A}_{TOC-tech}={A}_{investment-tech}+{A}_{FOC-tech}+{A}_{VC-tech}$$

10 $${A}_{investment-tech}={I}_{tech}\times \frac{i}{\left[1-{\left(1+i\right)}^{-n}\right]}$$

11 $${A}_{FOC-tech}={P}_{FOC-tech}\times {I}_{tech}$$

where i and n in Eq. (10) is the interest rate and lifespan of each power plant. Also, ${P}_{FOC-tech}$ is the annual fixed O & M costs.

## Results and discussions

The potential of solving the energy/electricity poverty challenge in sub-Sahara Africa with RE-based power plants has been analyzed in this study. The singular use and hybridization of solar PV, CSP, and onshore wind power plants, as well as pumped hydro storage, has been proposed in this study. Specifically, six different scenarios have been modeled and analyzed considering 2030 and 2040 as the targeted implementation years. To validate the accuracy of the model, existing literature has been reviewed. The EnergyPLAN model presented in this study and the data has been used in existing works of literature to evaluate the future energy system of different countries and regions including; Nigeria^88^, Middle East and North African region^89^, Algeria^90^, Burundi^91^, etc. Thereby validating the accuracy of the model for this analysis. Also, some of the dataset used in this study have been generated from Renewables.ninja and data generated from the platform has been used for different research projects^92–94^.

Based on the model developed in this study, the estimated electricity demand for the countries in this study is 678 TWh/yr for 2030 and 760 TWh/yr for 2040. Considering 22 TWh/yr and 40 TWh/yr BEV demand, the total electricity demand is 700 TWh/yr and 800 TWh/yr for 2030 and 2040 respectively. The hourly maximum and minimum electricity demand considering the one-year period are 117,167 MW and 39,630 MW while the average electricity demand is 77,186 MW.

**Figure 7:**
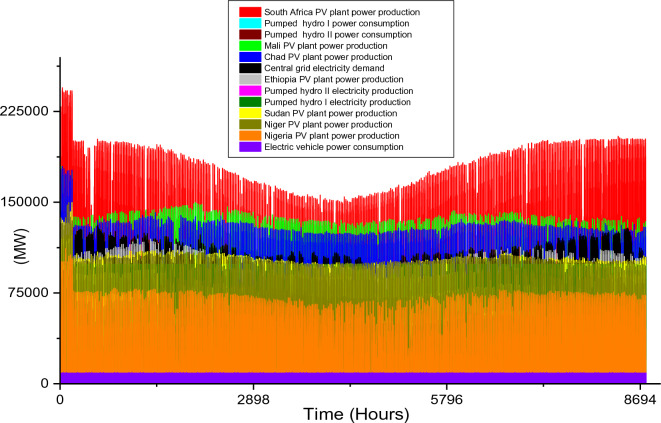
Solar PV case scenario electricity demand and supply for year 2040.

**Figure 8:**
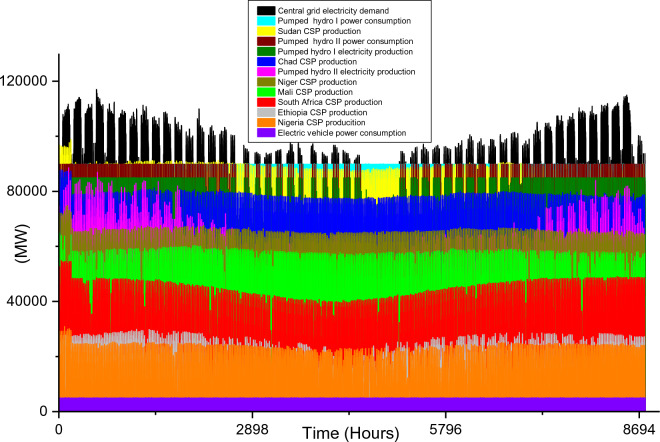
CSP case scenario electricity demand and supply for year 2030.

**Figure 9:**
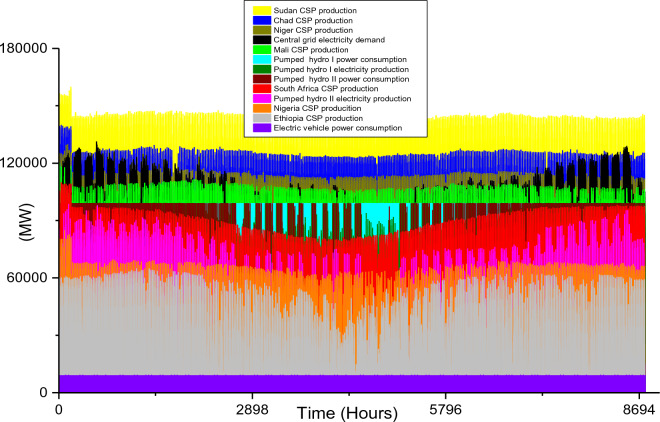
CSP case scenario electricity demand and supply for year 2040.

**Figure 10:**
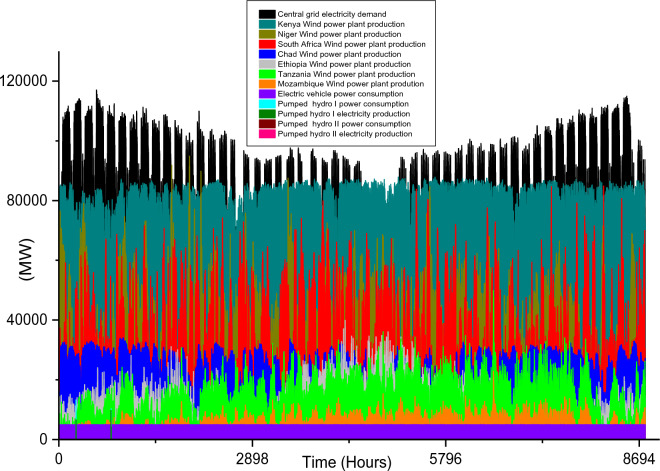
Wind power case scenario electricity demand and supply for year 2030.

**Figure 11:**
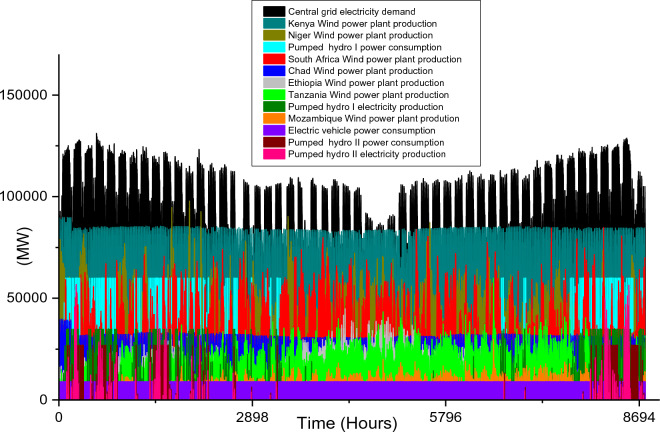
Wind power case scenario electricity demand and supply for year 2040.

**Figure 12:**
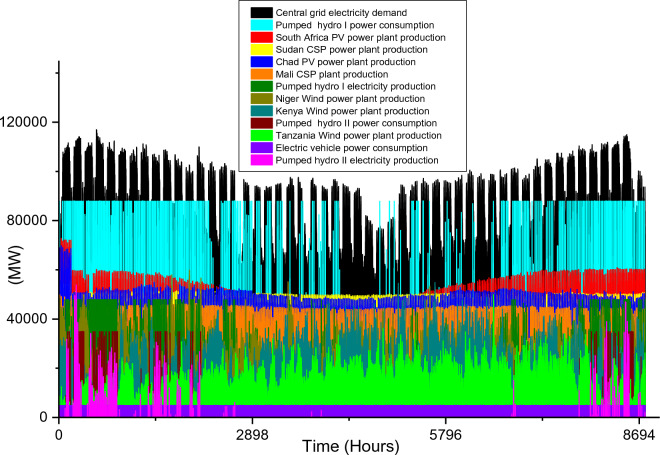
Hybrid with low storage case scenario electricity demand and supply for year 2030.

**Figure 13:**
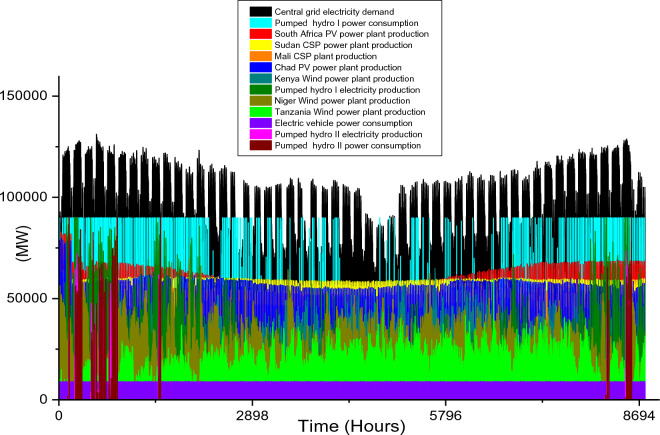
Hybrid with lor storage case scenario electricity demand and supply for year 2040.

Based on these estimated demands, the models are developed to meet the electricity requirements while observing the technical limitations of each RE-based technology. The results of the analysis are discussed in this section considering the single source and hybrid technologies. The analysis results highlight and summarize the generation capacities from different countries and technologies, the storage capacities, the economic commitments, and the electricity production. These are presented in Tables 5 and 6 for single and hybrid technologies respectively. While these tables (Tables 5 and 6) summarize the required renewable energy generation capacities (in MW) for each technology and country, the hourly production profile for a typical year from these technologies and countries are plotted and presented in Figs. 7, 8, 9, 10, 11, 12, 13.

**Table 5:** Simulation results summary for single technological scenarios.

Tech	Year	Generation capacities and location	Storage capacities	Total investment cost (10^9^ USD)	Total annual cost (10^9^ USD)	RE share in electricity produced (%)	Annual electricity production (TWh/yr)
PV	2030	Nigeria – 70,000 MW	Pump I – 90,000 MW	652	36.39	132.4	1316.57
		Sudan – 90,000 MW	Turbine I – 47,000 MW				
		Niger – 90,000 MW	Storage I – 700GWh				
		Mali – 100,000 MW					
		Chad – 100,000 MW	Pump II – 90,000 MW				
		Ethiopia – 80,000 MW	Turbine II – 89,000 MW				
		SA – 135,000 MW	Storage II – 750GWh				
	2040	Nigeria – 112,000 MW	Pump I – 99,000 MW	1078	59.17	189.4	2261.48
		Sudan – 150,000 MW	Turbine I – 99,000 MW				
		Niger – 150,000 MW	Storage I – 900GWh				
		Mali – 180,000 MW					
		Chad – 180,000 MW	Pump II – 99,000 MW				
		Ethiopia – 120,000 MW	Turbine II – 99,000 MW				
		SA – 245,000 MW	Storage II – 900GWh				
CSP	2030	Nigeria – 31,000 MW	Pump I – 90,000 MW	2780	378	118.5	1237.75
		Sudan – 99,000 MW	Turbine I – 85,000 MW				
		Niger – 75,000 MW	Storage I – 800GWh				
		Mali – 65,000 MW					
		Chad – 88,000 MW	Pump II – 90,000 MW				
		Ethiopia – 30,000 MW	Turbine II – 90,000 MW				
		SA – 55,000 MW	Storage II – 750GWh				
	2040	Nigeria – 85,000 MW	Pump I – 99,000 MW	4989	685	187.7	2229.29
		Sudan – 160,000 MW	Turbine I – 99,000 MW				
		Niger – 130,000 MW	Storage I – 850GWh				
		Mali – 120,000 MW					
		Chad – 140,000 MW	Pump II – 99,000 MW				
		Ethiopia – 65,000 MW	Turbine II – 99,000 MW				
		SA – 110,000 MW	Storage II – 700GWh				
Wind	2030	Mozambique – 23,000 MW	Pump I – 56,000 MW	517	40.65	252.6	1768.6
		Niger – 95,000 MW	Turbine I – 70,000 MW				
		Ethiopia – 40,000 MW	Storage I – 800GWh				
		SA – 85,000 MW					
		Kenya – 88,000 MW	Pump II – 39,000 MW				
		Chad – 34,000 MW	Turbine II – 71,000 MW				
		Tanzania – 50,000 MW	Storage II – 750GWh				
	2040	Mozambique – 30,000 MW	Pump I – 60,000 MW	533	42.59	155.7	1315.89
		Niger – 98,000 MW	Turbine I – 35,000 MW				
		Ethiopia – 45,000 MW	Storage I – 1000GWh				
		SA – 85,000 MW					
		Kenya – 90,000 MW	Pump II – 27,000 MW				
		Chad – 40,000 MW	Turbine II – 58,000 MW				
		Tanzania – 60,000 MW	Storage II – 1300GWh

**Table 6:** Simulation results summary for hybrid technological scenarios.

Tech	Year	Generation capacities and location	Storage/import capacities	Total investment cost (10^9^ USD)	Total annual cost (10^9^ USD)	RE share in electricity produced (%)	Annual electricity produced (TWh/yr)
Hybrid low storage	2030	Niger (Wind) – 60,000 MW	Pump I – 88,000 MW	1023	115	138.5	1126.32
		Kenya (Wind) – 55,000 MW	Turbine I – 48,000 MW				
		Tanzania (Wind) – 50,000 MW	Storage I – 800GWh				
		SA (PV) – 73,000 MW					
		Chad (PV) – 70,000 MW	Pump II – 35,000 MW				
		Mali (CSP) – 80,000 MW	Turbine II – 54,000 MW				
		Sudan (CSP) – 56,000 MW	Storage II – 750GWh				
	2040	Niger (Wind) – 70,000 MW	Pump I – 90,000 MW	1217	136.5	189.4	2261.48
		Kenya (Wind) – 65,000 MW	Turbine I – 90,000 MW				
		Tanzania (Wind) – 60,000 MW	Storage I – 950GWh				
		SA (PV) – 83,000 MW					
		Chad (PV) – 80,000 MW	Pump II – 90,000 MW				
		Mali (CSP) – 60,000 MW	Turbine II – 90,000 MW				
		Sudan (CSP) – 66,000 MW	Storage II – 800GWh				
Hybrid high storage	2030	Niger (Wind) – 45,000 MW	Pump I – 95,000 MW	870	94.33	97.1	868.37
		Kenya (Wind) – 40,000 MW	Turbine I – 49,000 MW				
		Tanzania (Wind) – 35,000 MW	Storage I – 6000GWh				
		SA (PV) – 60,000 MW					
		Chad (PV) – 57,000 MW	Pump II – 85,000 MW				
		Mali (CSP) – 39,000 MW	Turbine II – 57,000 MW				
		Sudan (CSP) – 45,000 MW	Storage II – 7500GWh				
	2040	Niger (Wind) – 55,000 MW	Pump I – 71,000 MW	980	105.76	103.2	1020.99
		Kenya (Wind) – 50,000 MW	Turbine I – 51,000 MW				
		Tanzania (Wind) – 45,000 MW	Storage I – 9500GWh				
		SA (PV) – 68,000 MW					
		Chad (PV) – 65,000 MW	Pump II – 36,000 MW				
		Mali (CSP) – 43,000 MW	Turbine II – 88,000 MW				
		Sudan (CSP) – 50,000 MW	Storage II – 7400GWh				
Hybrid with import power	2030	Niger (Wind) – 45,000 MW	Import from existing power plants – 106,000 MW	298	96.64	56.6	396.36
		Kenya (Wind) – 40,000 MW					
		Tanzania (Wind) – 34,000 MW					
		SA (PV) – 3000 MW					
		Chad (PV) – 4000 MW					
		Mali (CSP) – 2000 MW					
		Sudan (CSP) – 2500 MW					
	2040	Niger (Wind) – 54,000 MW	Import from existing power plants – 121,000 MW	439.34	107.44	61.8	494.46
		Kenya (Wind) – 50,000 MW					
		Tanzania (Wind) – 45,000 MW					
		SA (PV) – 3000 MW					
		Chad (PV) – 2000 MW					
		Mali (CSP) – 5000 MW					
		Sudan (CSP) – 3000 MW

### Single source RE power technologies

The use of solar PV to solve the energy poverty problem by 2030 will require a total of 665,000 MW installation in the seven different countries namely, Nigeria, Sudan, Niger, Mali, Chad, Ethiopia, and South Africa. Additionally, the pumped hydro storage capacity for the two reservoirs is 700 GWh for storage I and 750 GWh for storage II. While the capacity of the pumps required for the storage systems is the same (90,000 MW), the turbine capacities are 47,000 MW for storage I and 89,000 MW for storage II.

The huge PV plant capacities required to solve the imminent problem by 2030 are reflected in the total investment cost ($652 billion) and total annual cost ($36.39 billion). Also, these PV plants will produce 926.8 TWh/yr of electricity which is 132.4% of the required electricity demand. It is noteworthy that the two pumped hydro storage systems will produce a total of 338 TWh/yr and the expected yearly CEEP when this technology is adopted is 484.76 TWh/yr. The BEVs integrated with the proposed RE central grid system will consume 5009 MW of electricity hourly for 12 h daily and this is constant for all the single RE power plants considering the year 2030. The hourly electricity demand/consumption and production profile year 2030 is illustrated in Fig. 6.

For the additionally 100 TWh/yr electricity demand in 2040 (800 TWh/yr), the 2030 PV plants capacities will increase by 42,000 MW for Nigeria, 60,000 MW for Sudan and Niger, 80,000 MW for Mali and Chad, 40,000 MW for Ethiopia and 90,000 MW for South Africa. This also increases the total investment cost and annual cost by $426 billion and $22.78 billion. The total electricity produced by the PV plants is 189.4% of the electricity demand.

The unavailability of solar radiation during the night hours creates a wide gap between the electricity demand and production. Although the economic per unit cost of PV plant is relatively the cheapest of all the technologies considered in this research, the huge installation capacities and CEEP is an impeding factor that must be overcome. The electricity demand and consumption profile for 2040 (illustrated in Fig. 7) show the nature/potency of solar energy in SSA. This also highlights the huge increase in generation capacities when compared with the year 2030 (in Fig. 6). Also, the total hydrogen that will be generated from the CEEP in the central RE grid is 9.21 × 10^9^ kg/yr for 2030 and 2.5 × 10^10^ kg/yr for 2040.

The countries selected for solar PV plant installation are the same as the countries selected for CSP plant installation, however, the installation capacity distribution is different. Since solar PV performs better when the solar temperature is not extremely high, South Africa with moderate solar temperature was considered to dominate solar PV plants. On the other hand, Chad will dominate CSP plant installation due to its enormous solar potential for this specific technology. The total installed capacity to meet the 2030 electricity demand for all the countries considered is 443 MW and the specific distribution of this for each country is presented in Table 5. Although this is 222 MW lower than its corresponding solar PV, the high economic cost of CSP plants results in the highest total investment ($2.78 trillion) and total annual costs ($378 billion) when compared with the three single source technologies. The 118.5% CSP share in total electricity produced shows that the power plants will require a sizable amount of storage to ensure they meet the 2030 electricity demand. As seen in Table 5, the pumped hydro storage capacities are 800 GWh storage reservoir, 90,000 MW pump, and 85,000 MW Turbine for storage I and 750 GW storage, 90,000 MW pump, and 90,000 MW Turbine for storage II. The total electricity produced by this system amounts to 1237.75 TWh/yr and its hourly distribution is plotted in Fig. 8. The total hydrogen production is 7.76 × 10^9^ kg/yr.

To meet the energy demands in 2040, there will be a huge increase in the CSP plants' installation capacities in 2030. The pumped hydro storage pump and turbine requirements are identical to that of the year 2040 PV plants case scenario; however, the storage reservoir will differ (850 GWh for storage I and 700 GWh for storage II). The required CSP capacities for 2040 for each country are highlighted in Table 5 and the electricity demand/consumption and production for all the technologies and the storage systems are illustrated in Fig. 9. In comparison to Fig. 8, it is obvious that the production of the CSP plants in Sudan, Niger, and Chad are higher than the required electricity by the central grid for some hours during the year. This is because of the unavailability of solar energy during the night hours. The huge power plant capacities are also evident in the total electricity production over a period of one year. Although the required electricity demand is 800 TWh/yr, the CSP plants and the storage systems will have a total electricity production of 2229.29 TWh/yr (out of which 514. 41 TWh/yr is consumed by the storage systems) and net electricity production of 1714.88 TWh/yr. The hydrogen produced from the CEEP in the central grid is enormous (2.44 × 10^9^ kg/yr). As seen in Fig. 9, the hourly electricity consumption by the BEVs connected to the central grid in 2040 is 9107 MW, and this is constant for the 12-h charging periods. Also, this is the same for all the 2040 case scenarios in this research.

Considering these technical and economic analysis results, the use of solar-based technologies (PV or CSP) to meet the electricity requirement seems sensible for 2030. However, the electricity production, CEEP, and economic costs show that the singular use of PV or CSP has a very low economic and technical feasibility. Hence the analysis of onshore wind farms for power generation in this study.

Unlike solar energy that is unavailable at night, wind power plants are plagued with the stochastic nature of wind energy, and this causes a rather fluctuation in the wind farm production. In comparison to the singular use of the two solar energy technologies, the adoption of onshore wind farms requires the least total installation capacities for both 2030 (which is 415,000 MW) and 2040 (which is 448,000 MW) case scenarios. The countries considered for the onshore wind farm installation include Mozambique, Niger, Ethiopia, South Africa, Kenya, Chad, and Tanzania. The specific capacities for each country’s wind farm are shown in Table 5. It is also notable that the gap in required power plant capacities for 2030 and 2040 case scenarios is much lesser when compared to solar-based technologies.

The onshore wind farms will only require a 33,000 MW increase in order to meet the central grid electricity demand in 2040. The electricity demand and production profiles plotted in Figs. 10 and 11 show that Kenya’s wind power production is paramount to the stability of the central grid system. This also highlights enormous wind potential in this country thereby confirming the results in existing studies^95,96^.

In summary, the singular use of onshore wind power technology is the most feasible solution for 2030 and 2040 case scenarios if SSA will adopt the use of a singular RE technology to solve its energy poverty problem. Considering the economic costs, the $517 billion total investment cost for the year 2030 is $135 billion cheaper than solar PV technology and almost half of CSP cost. Similarly, for 2040 case scenario, the $533 billion investment cost is about half that of solar PV technology and it is almost 10 times cheaper than CSP technology (Table 5). Although the total annual cost for the 2030 solar PV case scenario is $4.26 billion cheaper than its corresponding wind onshore case, the huge difference in the total investment cost is an undeniable advantage for wind power. The $42.59 billion total annual cost is also the cheapest of all the singular technology scenarios for 2040 (Fig. 12).

The installation capacities and their corresponding electricity production by the wind farm are also high and this further agrees with the current incremental adoption of wind power globally. Although the two storage reservoir’s capacities (1000 GWh and 1300 GWh for storage I and II respectively) are relatively higher than other case scenarios, the relatively lower pump, and turbine capacities complement the high reservoir capacities. The use of wind onshore power plants leads to comparatively smaller CEEP for 2030 and 2040 case scenarios which are reflected in the total annual electricity production (in Table 5). Hence the total yearly hydrogen production (2.03 × 10^10^ kg/yr for 2030 and 9.52 × 10^9^/yr kg for 2040).

### Hybrid RE power technologies

Although the use of single RE technology (as explained in Sect. 4.1) has the potential of solving the energy poverty in SSA, the energy security and stability issue attached to the use of single technology RE power system is still a source of concern globally. In the existing literature, the hybridization of RE technologies has been proposed as the potent pathway to a net-zero emission sustainable global future. Therefore, the hybrid of onshore wind, PV, and CSP is also analyzed in this study to meet the 2030 and 2040 electricity demand in the selected countries in SSA.

**Figure 14:**
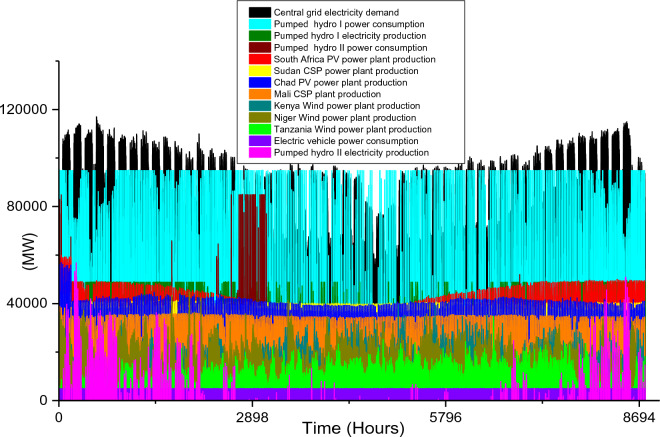
Hybrid with high storage case scenario electricity demand and supply for year 2030.

**Figure 15:**
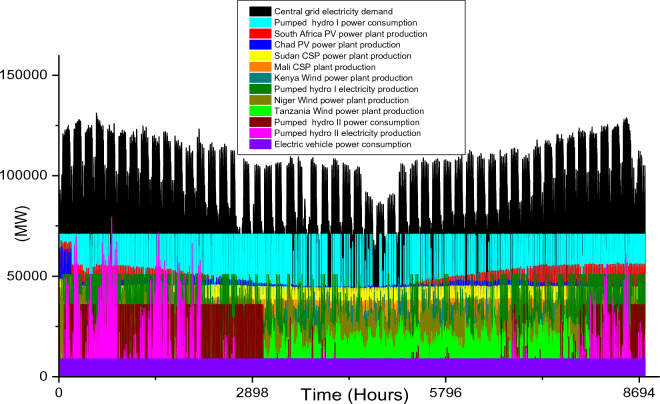
Hybrid with high storage case scenario electricity demand and supply for year 2040.

**Figure 16:**
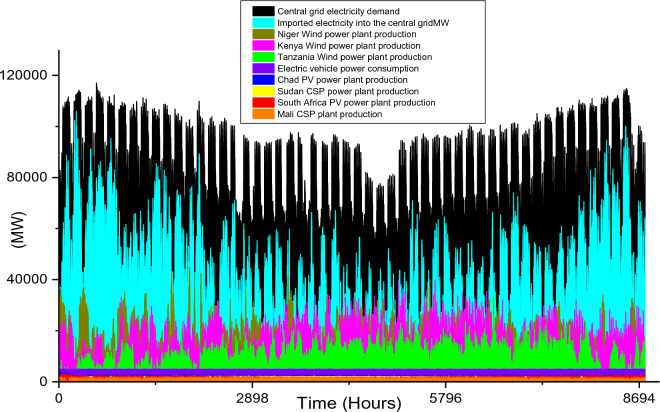
Hybrid with import case scenario electricity demand and supply for year 2030.

While Niger, Kenya, and Tanzania are considered for onshore wind power technology, South Africa and Chad/Mali and Sudan are considered for PV/CSP technologies respectively. Also, three hybrid scenarios are analyzed. In comparison to all the single RE technologies scenarios, the total installation capacities required for the hybrid scenarios are smaller (except for the wind power 2030 scenario). The required power plant capacities for each technology in different countries are highlighted in Table 6. Also, the electricity demand and production hourly profile for all the scenarios are illustrated in Fig. 13, 14, 15, 16, 17. The utilization of a hybrid RE power plant with low storage resulted in high CEEP in the power system as the RE share in the total electricity produced is 138.5% and 189.4% for 2030 and 2040 electricity demands. Therefore, the hydrogen produced from the CEEP in 2030 and 2040 case scenarios is 7.5 × 10^9^ kg/yr and 9.16 × 10^9^ kg/yr.

**Figure 17:**
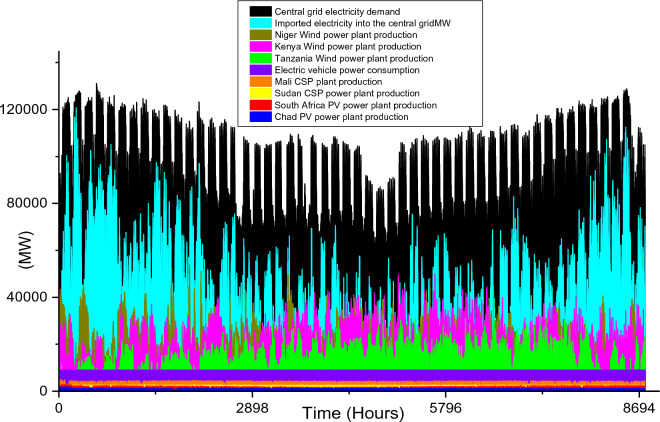
Hybrid with import case scenario electricity demand and supply for year 2040.

Although these hybrid scenarios consider the minimum pumped hydro storage required, the storage consume a sizable quantity of electricity to augment the RE power plants' production (as illustrated in Fig. 12). Also, the annual electricity production considering the use of a hybrid power system with low storage capacity is 1126.32 TWh/yr for 2030 and 2261. TWh/yr for 2040 (Table 6). While the electricity consumption by the storage system for the 2040 hybrid with low storage case scenario is high, the electricity production by these storages are high (Fig. 13). The electricity production in 2040 is 2.77 times the required capacity, thereby increasing the economic parameters.It is noteworthy that the $1023 billion and $1217 billion total investment costs for the 2030 and 2040 hybrid low storage scenarios are the highest investments cost of all the hybrid scenarios.

Considering a decarbonized carbon emission power system, the integration of high storage with the hybrid power plants proved to be the most feasible solution of all the scenarios in this study. For these case scenarios, the total optimal power plant capacities are 321,000 MW for 2030 and 376,000 MW for 2040. These are significant reductions to their corresponding scenarios for the hybrid with low storage cases. However, the total reservoir storage capacities for these scenarios (13,500 GWh for 2030 and 16,900GWh for 2040) are 8 times or more of its corresponding low storage scenarios. The high storage reservoirs are reflective on the power consumption by the storage pumps as illustrated in Fig. 14 and Fig. 15. In these scenarios, the RES share in the production and the annual electricity is almost the same as the demands, thereby reducing the CEEP significantly. The corresponding amount of hydrogen producible from the CEEP is 2.07 × 10^9^ kg/yr for 2030 and 3.02 × 10^9^ kg/yr for 2040. Based on this high storage integration, there is a significant reduction in the total annual and investment cost of the high storage hybrid system when compared with low storage hybrid systems (as seen in Table 6).

Acknowledging that all the countries selected for the simulation in this study have an existing power system in place currently, it is important to also model a scenario that can accommodate the integration of existing power systems (fossil fuel power plants) with the hybrid RE scenarios. The optimal hybrid power plant capacities for 2030 estimated electricity demand include 45,000 MW of wind power in Niger, 40,000 MW of wind power in Kenya, 34,000 MW of wind power in Tanzania, 3000 MW PV plant in South Africa, 4000 MW PV plant in Chad, 2000 MW CSP plant in Mali, and 2500 MW CSP plant in Sudan. In addition, the import of 106,000 MW of electricity is required in the central grid from the existing or fossil-fueled power plants of these countries to achieve 100% load stability (Table 6). Therefore, the electricity produced (396.36 TWh/yr) by the hybrid RE power plants is 56.6% of the total electricity required. For this case scenario, there is no CEEP as the RE power plant production is maximized when available. The total electricity imports to the RE central grid is 305.37 TWh/yr.

As illustrated in Fig. 16, electricity importation into the grid is relatively high in January, February, March, and December as the monthly average electricity import for these months is 53,332 MW, 46,103 MW, 47,740 MW, and 50,363 MW respectively. These months coincide with months of relatively low wind power and solar power potential for Kenya, Tanzania, and Mali, thereby having a significant effect on the hybrid RES production. The economic costs of this scenario (Table 6) are significantly lower in comparison to other case scenarios in this study. This can be attributed to large electricity imports into the grid system. It is worth noting that the imported electricity is assumed to be from existing power plants, therefore the investment cost for these systems is zero (although the operation and maintenance costs are considered).

For the 2040 estimated electricity demand, 15,000 MW of electricity is imported into the central grid in addition to the 2030 electricity import from existing power systems. The RES power plants will also require upgrades in capacities (Table 6); however, the upgrade is minimal in comparison to other scenarios in this study (especially for solar-based power plants). While the total investment cost ($439.34 billion) is smaller than other case scenarios for 2040, the annual investment cost which includes the yearly operation and maintenance cost is comparable to other scenarios. In theory, this scenario provides the fastest and the most feasible pathway to achieving 100% electrification in the selected countries in SSA. Also, there will be no hydrogen production from this case scenario, although the integration of electric vehicles is considered based on the pre-defined capacity with the grid. The hourly electricity demand/consumption and production profile for this scenario is plotted in Fig. 17 and the high electricity importation from the existing power plants is evident in this diagram.

The optimal feasibility of the central grid system in SSA is achieved using the hybrid power plants as retrieved in this study. The comparative advantage of technical and economic optimality of the hybrid energy system will be because of the strategic location of the hybrid power facilities, the efficient use of renewable energy sources, and the incorporation of storage technologies (references). To optimize the viability of the central grid system in Sub-Saharan Africa (SSA), the strategic placement of hybrid power facilities is essential. Ishaku et al.^97^ conducted a central grid analysis for the West African Power Pool (WAPP) that corroborates this idea. By strategically situating hybrid power plants in suitable locations throughout the region, it is possible to maximize the use of renewable energy sources in those areas. Wind energy will play a huge role in the SSA central grid, as shown in this work. The study by Obadia et al.^43^ stated the Makambako (Njombe) and Kititimo (Singida) in Tanzania are being set aside for huge wind generation. Several studies have also shown the high renewable energy of the SSA countries as used in this study, and their technical feasibility. Conclusively, it is worth stating that some bottlenecks like restraining factors deficiencies in regional institutions and at the government level, financial scarcity, inadequate infrastructure, a lack of interconnection between member states, and a deficient skill base for the integration of renewable energy could hinder SSA regional electricity trade, hence policy decision to facilitate its growth is important^97^.

### Greenhouse gas emission savings

With the exception of the hybrid with import power case scenarios, all the proposed pathways in this study will have no carbon emission from electricity production. Since the electricity demand for all the scenarios is the same, the greenhouse gases saved yearly when compared with #6 fuel oil (the emission parameters are presented in Table 4) will be the same. Therefore, the greenhouse gases emission savings from the implementation of this technology is illustrated in Table 7. Since the source of the electricity imported into the grid is not critically analyzed, fossil fuels are assumed for these scenarios (Table 7). This enormous greenhouse gas emission reduction potential further shows the need for the implementation of this study as the quantity of CO2 saved yearly can be as much as 1.04 × 10^11^ kg by 2030 and even increase to 1.30 × 10^11^ kg by 2040.

**Table 7:** Greenhouse gases emission reduction potential.

Greenhouse gas emission savings	Hybrid with import power case scenario		Other case scenarios	
	2030	2040	2030	2040
CO2 (kg/yr)	104,000,000,000	130,000,000,000	184,100,000,000	210,400,000,000
NO_X_ (kg/yr)	294,495,480	367,383,780	520,100,000	594,400,000
SO_X_ (kg/yr)	582,649,200	726,856,200	1,029,000,000	1,176,000,000

## Conclusions

In this study, the potential of RE is juxtaposed against the electricity poverty in SSA. The prospect of using wind power, solar PVs, and CSP to tackle the impending/imminent electricity challenge (energy poverty) in this continent is analyzed. A central grid is proposed for 12 selected countries and the electricity demands of these countries are estimated for the years 2030 and 2040. The aforementioned RE technologies are considered for installation in seven different countries for each case scenario while the remaining countries were considered for two pumped hydro storage installations. Beyond the use of RES only, the integration of the existing power systems in these countries with new RE power plants was also considered. A total of 12 different solution scenarios to the energy poverty in SSA was proposed and analyzed. The key conclusions from this study include:

- Although the RE potential in this region is enormous, the review of the energy poverty in SSA showed that the electricity access of all the countries (except for Seychelles) in this region is less than 100%.
- The analysis of the proposed central RE grid system is a viable and sustainable option to solve this energy poverty in SSA. However, strategic planning will be required to raise money for the implementation. The cheapest investment cost from all the case scenarios in this study is $298 billion. However, this scenario will involve a power import capacity of 106,000 MW from existing power plants. Also, the green energy capacity from different locations is 45,000 MW of wind from Niger, 45,000 MW of wind from Kenya, 34,000 MW of wind from Tanzania, 3000 MW of solar PV from South Africa, 4000 MW of solar PV from Chad, 2000 MW of CSP from Mali, and 2500 MW of CSP from Sudan. It can be concluded that the integration of existing green power plants across Africa will significantly reduce the overall total investment cost.
- Considering the use of a single RE technology to solve the electricity problem, the implementation of wind power systems by 2030 and 2040 are the most feasible options as they have the least CEEPs and economic costs. Although solar PV analysis for 2030 records the least annual investment cost, the total investment cost of this case scenario makes wind power systems a more preferable option.
- To have energy security and achieve a decarbonization from electricity production by 2030 or 2040, the “Hybrid with High Storage” case scenario is most the viable option.
- Overall, the integration of the existing power systems with new RE technologies for the proposed central grid (“Hybrid with Import Power” case scenario) will be the cheapest and easiest pathway as it requires the least economic commitments. Also, this scenario does not require the use of storage systems, and this will help the SSA countries reduce the carbon emission from their electricity sector by 56.6% and 61.8% by 2030 and 2040 respectively.

The utilization of the proposed solutions in this study may require the help of global energy organizations or the world bank. Also, the development/implementation of strategic policies to encourage citizens to install these RE power systems will reduce the investment and annual cost burden on the government. It is noteworthy that the RE technologies in this study can be installed centrally or in smaller units in different parts of each country. Also, the model presented in this study can be applied to other continents and regions of the world with similar energy poverty challenges. Future research can focus on the integration of all the countries in SSA with the central RE grid systems.

## Data Availability

The datasets used and/or analyzed during the current study are available from the corresponding author on reasonable request.
